# Clinical verification of plasma messenger RNA as novel noninvasive biomarker identified through bioinformatics analysis for lung cancer

**DOI:** 10.18632/oncotarget.16701

**Published:** 2017-03-30

**Authors:** Dan Zhou, Weiwei Tang, Xinli Liu, Han-Xiang An, Yun Zhang

**Affiliations:** ^1^ Department of Medical Oncology, The First Affiliated Hospital of Xiamen University, Xiamen, Fujian, China; ^2^ Xiamen Institute of Rare Earth Materials, Chinese Academy of Sciences and Key Laboratory of Design and Assembly of Functional Nanostructures, Fujian Provincial Key Laboratory of Nanomaterials, Fujian Institute of Research on the Structure of Matter, Chinese Academy of Sciences, Xiamen, Fujian, China

**Keywords:** lung cancer, biomarker, cell-free RNA, HJURP, ADAMTS8

## Abstract

Lung cancer (LC) remains associated with significant mortality worldwide. The lack of reliable noninvasive biomarkers and targeted therapies contributes to poor survival rate. Herein, we initially took advantage of the public microarray data from Oncomine database to filter messenger RNAs (mRNAs) as potential biomarkers. Subsequently, clinical validation was applied to identify candidate noninvasive biomarkers in plasma from patients with LC. Through comprehensive analysis of transcriptional expression profiles across 12 studies, top 6 over- and underexpressed mRNAs were generated. Then, a pair of matched plasma samples from LC patient and normal control was detected by RT-PCR, and three genes with positive bands were selected for further validation. Finally, qPCR was conducted to further assess values of the three identified genes. We displayed with high confidence that two cell-free mRNAs (HJURP and ADAMTS8) were expressed at significantly levels compared to normal controls. Receiver-operating characteristic (ROC) curves on the diagnostic efficacy of plasma HJURP and ADAMTS8 mRNAs in LC diagnosis showed that the area under the ROC (AUC) was 0.6960 and 0.6877; sensitivity was 66.0% and 83.7%; specificity was 78.6% and 71.4%, respectively. Combined ROC analyses using these two biomarkers revealed an elevated AUC of 0.75. Furthermore, the higher HJURP level could be associated with early-stage LC while lower ADAMTS8 level could be correlated with non-small cell lung cancer. Collectively, circulating HJURP and ADAMTS8 mRNAs are promising noninvasive biomarkers for LC diagnosis. Our integrative strategy provides new insights into novel noninvasive biomarker identification for other types of cancer.

## INTRODUCTION

Lung cancer (LC) is currently ranked as the most common cancer and the leading cause of cancer-related mortality in males worldwide with roughly 1.8 million new cases diagnosed in 2012 (13% of all cancers) [1]. It is well established that LC is a clinically and pathologically heterogeneous disease and has been categorized into two main histological types non-small cell lung cancer (NSCLC) including squamous cell carcinoma, adenocarcinoma as well as large cell carcinoma and small cell lung cancer based on the origin of epithelial-cell precursors [2]. Risk factors including exposure to environmental and occupational carcinogens, have been associated with an increased incidence of LC [3]. The overall survival rate of LC patients is remarkably improved if diagnosis is confirmed at early stages, but limited access to effective screening with X-ray or computed tomography remains a problem [4]. Accordingly, identification of novel biomarkers for better diagnosis and for gaining insights into the molecular makeup of LC would be beneficial.

There is a growing interest in cell-free nucleic acids isolated from body fluids as diagnostic indicators for cancer pathology [5]. Circulating cell-free messenger RNA (mRNA) containing tumor-associated genetic alterations were first demonstrated in the 1990s in the plasma of patients with nasopharyngeal carcinoma [6]. A few years later, the investigation was expanded to patients with different type of cancers including breast cancer, colorectal cancer and lung cancer [7–9]. Collectively, these findings provide opportunities for cell-free mRNA to be served as appealing non-invasive biomarker candidates in various cancers including lung cancer. However, until now there have been only limited studies on the identification of cell-free mRNA level and the correlation between the presence of cell-free mRNA and the clinicopathological characteristics of LC patients.

One potential approach to obtain useful candidate biomarkers is the large body of publicly available microarray data [10]. The origin of circulating nucleic acids has been shown to be released from apoptotic and necrotic cancer cells as well as tissues [11]. Thus, we can collect a series of potential biomarkers on the basis of publicly tissue microarray data. In this study, we first took advantage of the large set of existing microarray to analyzed mRNA expression levels in LC tissues from Oncomine database via a cancer vs. normal analysis yielding a list of candidate mRNAs differentially expressed in LC. This was followed by a clinical validation study with multiple patient plasma samples that ultimately led to confirmation of several cell-free mRNAs as novel noninvasive biomarkers.

## RESULTS

### Identification of candidate mRNAs from Oncomine database

**Figure 1:**
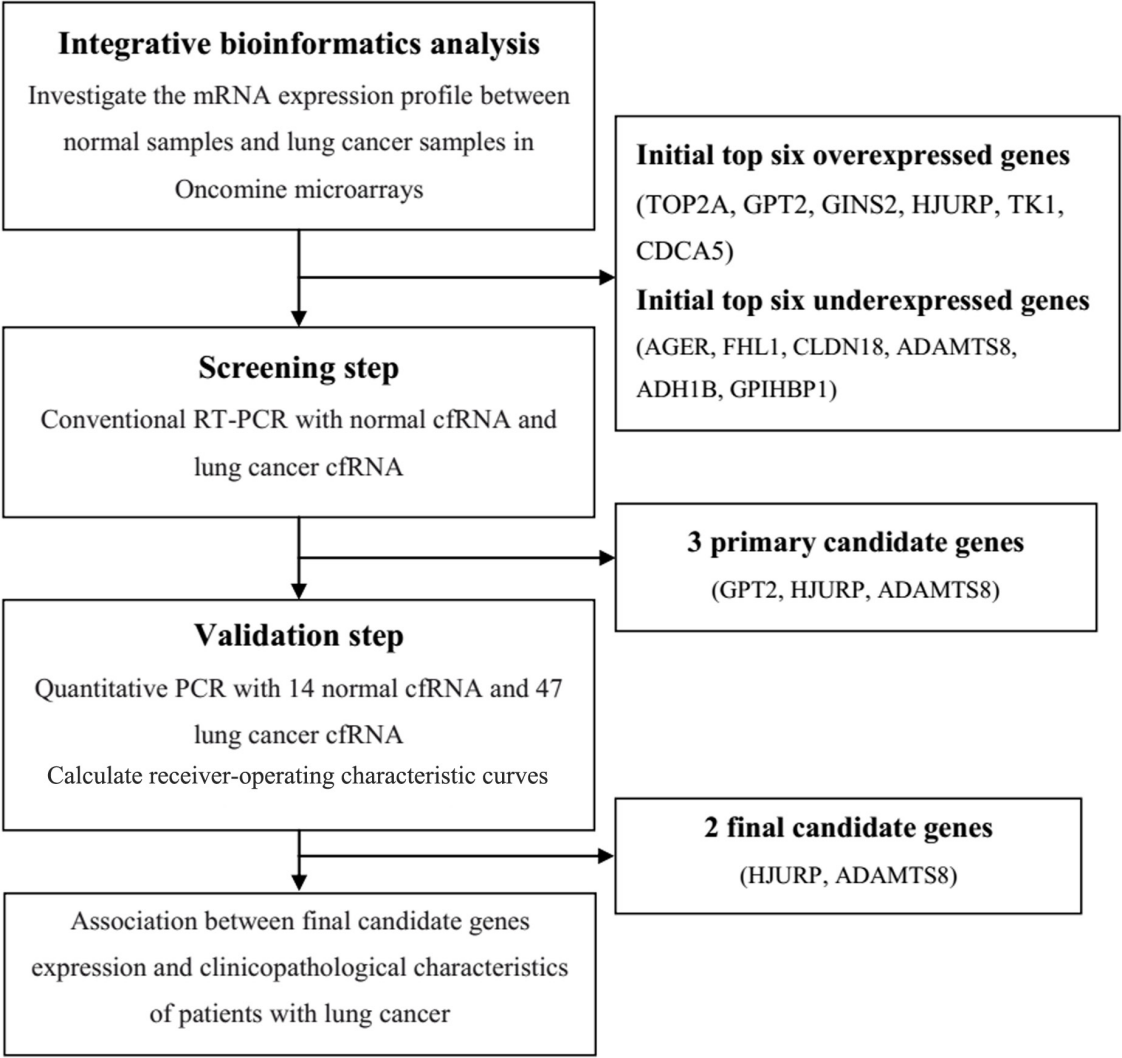
Schematic diagram of the overall procedure

**Figure 2:**
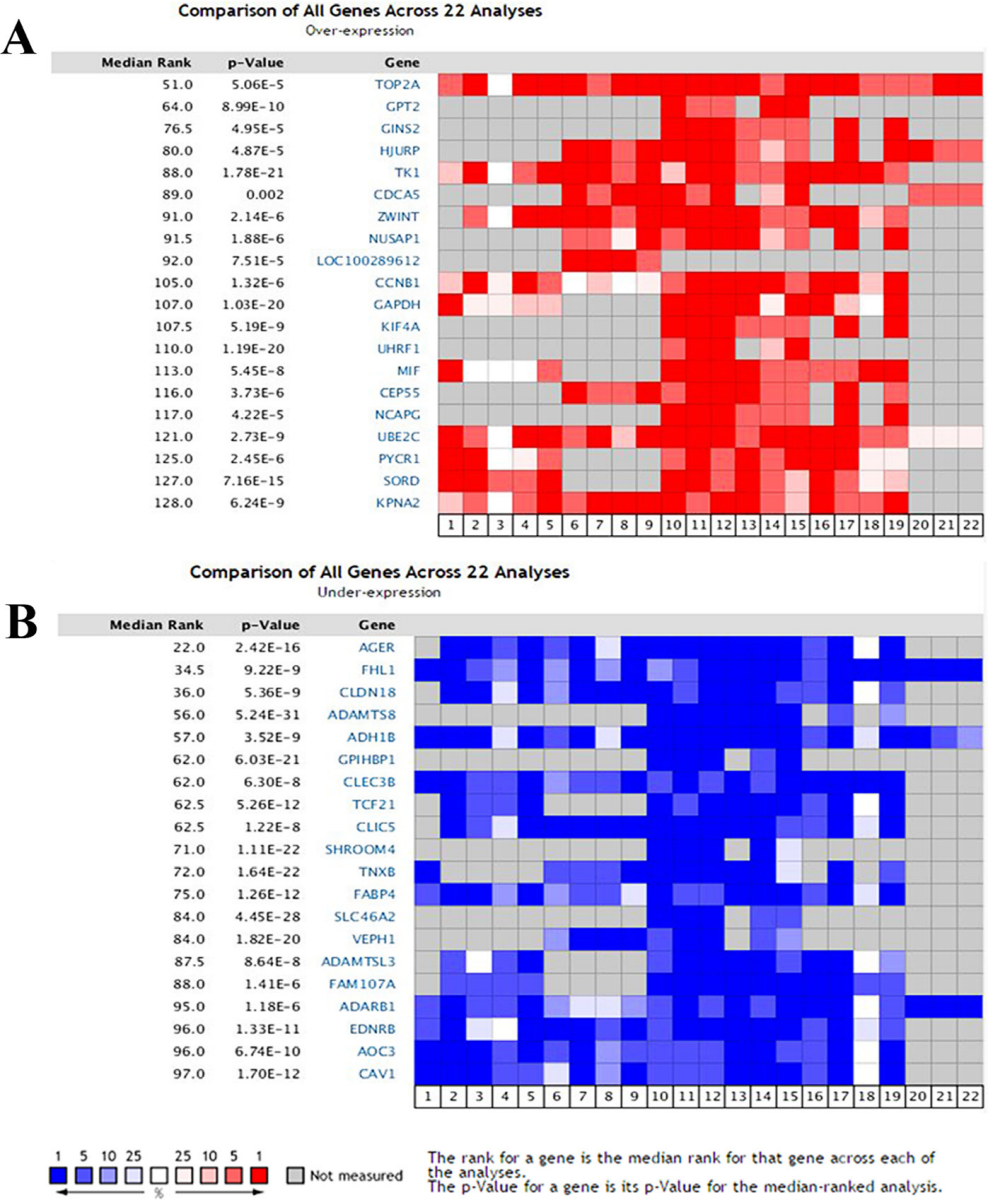
Transcriptional heat map of the top 20 over- and underexpressed genes in lung cancer samples compared with the normal samples through Oncomine analysis The level plots depict the frequencies (%) of **(A)** over- and **(B)** underexpressed candidate genes in 22 analyses from 12 included studies. Red cells represent overexpression. Blue cells represent underexpression. Gray cells represent not measured.

Using the Oncomine database we compared the mRNA expression level in lung cancer vs. normal according to a flow chart in Figure 1. In total, 892 lung cancer samples including 714 adenocarcinoma samples, 114 squamous cell lung carcinoma samples, 30 large cell lung carcinoma samples, 22 lung carcinoid tumor samples and 12 small cell lung carcinoma samples, along with 285 normal samples from 12 pioneering works on lung cancer were analyzed [12–23]. As shown in Figure 2, the Oncomine data yielded the following top ten overexpressed mRNAs: Top2A, GPT2, GINS2, HJURP, TK1, CDCA5, ZWINT, NUSAP1, LOC100289612 and CCNB1. Similarly, another set of the top ten underexpressed mRNAs resulted from the Oncomine analyses: AGER, FHL1, CLDN18, ADAMTS8, ADH1B, GPIHBP1, CLEC3B, TCF21, CLIC5 and SHROOM4.

In general there is concordance between the tumor-associated genetic alterations in plasma and those changes in the primary tumor. However, exceptions to this have also yielded. This implies that experimental validation of candidate biomarkers in cell-free mRNA (cfRNA) derived from analysis of the tissue microarray data using well-defined plasma samples is an essential step for reliably indentifying noninvasive biomarkers.

### Experimental validation of the candidate noninvasive biomarkers

As a result of the limited amount of the cfRNA, we selected 12 genes including top six overexpressed and underexpressed mRNAs for experimental validation. The validation procedures contained two procedures. Initially, conventional RT-PCR analysis was carried out for the 12 candidate mRNAs using a pair of matched plasma samples from LC patient and normal control. Three mRNAs were selected according to the existence of RT-PCR bands. Then, qPCR experiments were conducted for the three genes using cfRNA samples from 47 lung cancer patients and 14 healthy subjects.

### Conventional RT-PCR results

**Figure 3:**
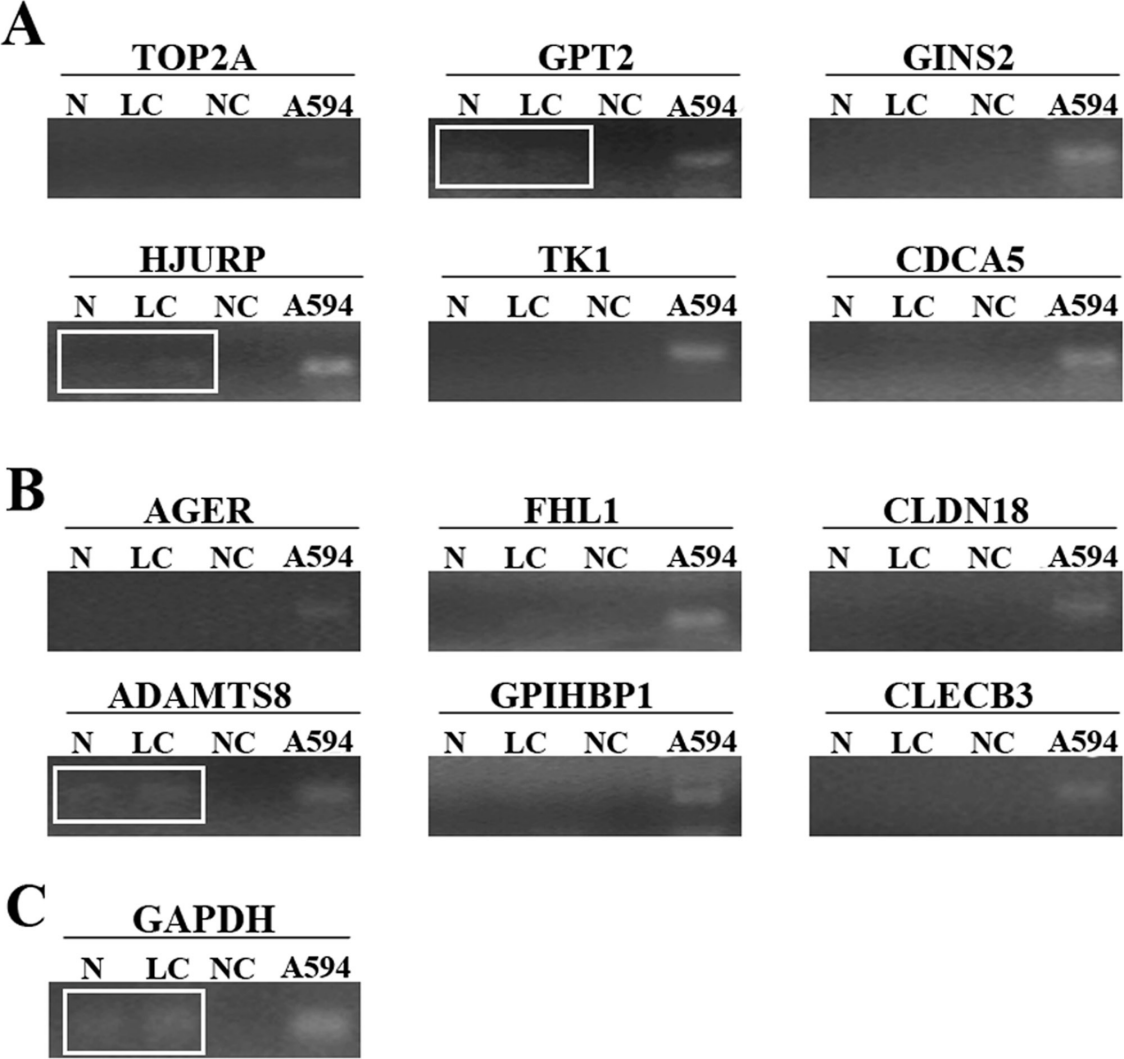
The amplified fragments of RT-PCR for top six overexpressed **(A)** including TOP2A, GPT2, GINS2, HJURP, TK1, and CDCA5 and underexpressed **(B)** including AGER, FHL1, CLDN18, ADAMTS8, ADH1B, and GPIHBP1) mRNAs. Weakly positive bands of GPT2, HJURP and ADAMTS8 were identified in cfRNA samples from lung cancer patient and healthy subject after separation in an agarose gel respectively. **(C)** GAPDH was amplified as the control PCR product. N represents cfRNA from healthy subject. LC represents cfRNA from lung cancer patient. NC represents no template control. A594 represent total RNA from A594 cells as positive control.

We employed conventional RT-PCR to examine 12 mRNAs expression including top six overexpressed (TOP2A, GPT2, GINS2, HJURP, TK1, and CDCA5) and underexpressed (AGER, FHL1, CLDN18, ADAMTS8, ADH1B, and GPIHBP1) mRNAs from the Oncomine analyses. As shown in Figure 3, 9 genes (TOP2A, GINS2, TK1, CDCA5, AGER, FHL1 CLDN18, ADH1B, and GPIHBP1) were excluded from further validation efforts due to the absence of the RT-PCR bands compared with positive control. In contrast, weakly positive bands of GPT2, HJURP and ADAMTS8 were observed in cfRNA samples from the healthy subject and matched LC patient in accordance with positive control. Taken together, these results indicated that GPT2, HJURP and ADAMTS8 might be closely associated with LC.

### QPCR results

To further validate transcriptional profiles of GPT2, HJURP and ADAMTS8 in LC, qPCR was conducted on cfRNA samples from 47 LC patients and 14 healthy subjects.

**Figure 4:**
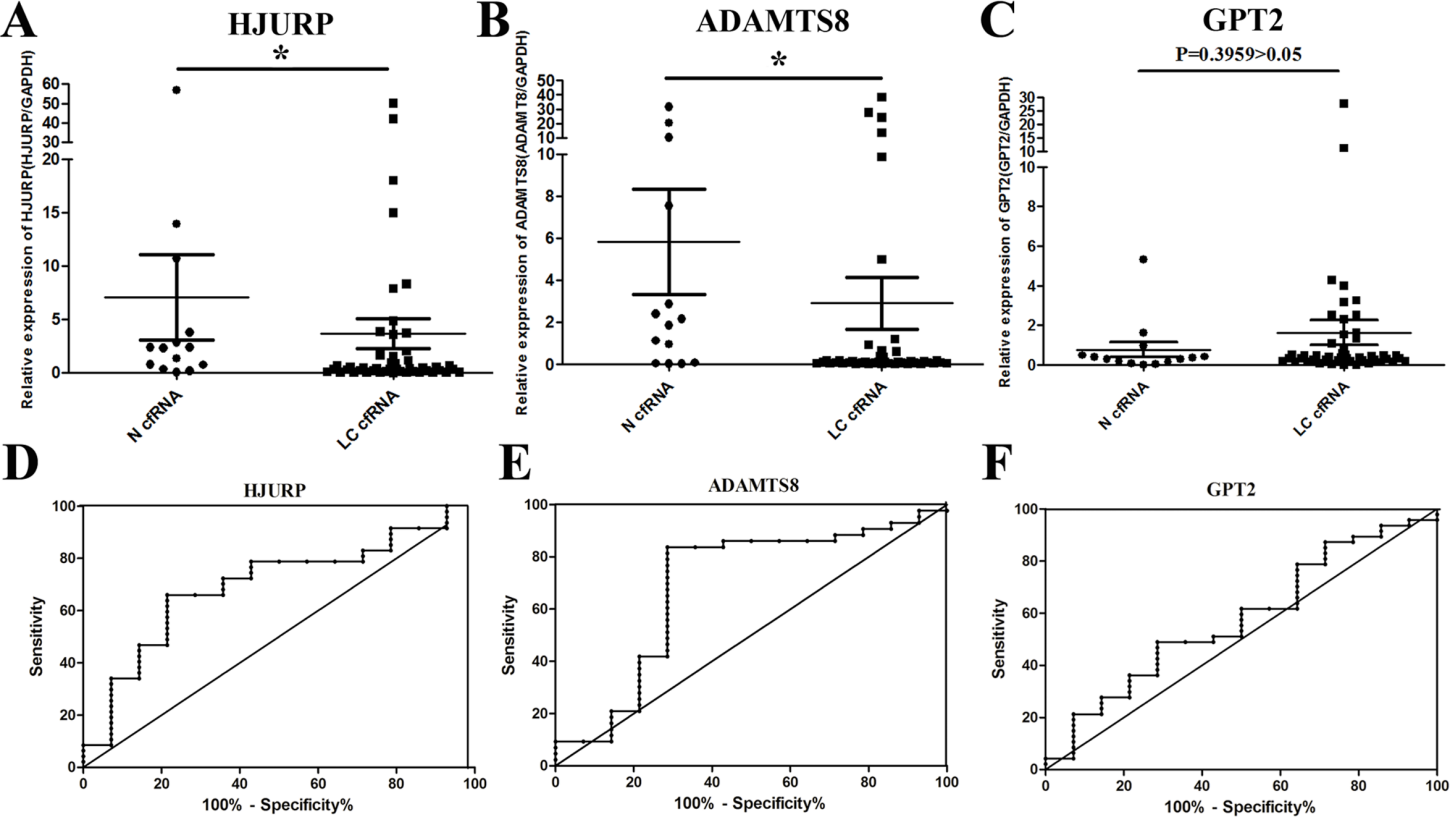
Clinical validation of candidate noninvasive biomarkers The change in circulating mRNA levels of HJURP **(A)**, ADAMTS8 **(B)** and GPT2 **(C)** mRNAs between lung cancer patients and normal subjects detected by qPCR. Data was shown as scatter plots and the intersecting line represents the median value with the interquartile range. Receiver-operating characteristic curves (ROC) showed the performances of fold-change in HJURP **(D)**, ADAMTS8 **(E)** and GPT2 **(F)** mRNA expression in predicting the lung cancer. Results were shown with means ± SEM. *p<0.05.

The expression of HJURP and GPT2 mRNA was detected in all LC plasma samples, whereas the expression of ADAMTS8 mRNA could only be detected in 43/47 LC samples. As shown in Figure 4A, 4B and 4C, the change in the expression of HJURP (p=0.0275) and ADAMTS8 (p=0.0370) but not GPT2 (p=0.3959) was significantly different between the LC cfRNA samples and healthy controls. In addition, the diagnostic efficacy of plasma HJURP and ADAMTS8 mRNAs in LC diagnosis was performed using receiver-operating characteristic (ROC) curves. At the optimal cut-off (<0.7104), HJURP had a 66.0% sensitivity and a 78.6% specificity in separating LC patients from healthy subjects with an AUC of 0.6960 (Figure 4D). Similarly, as shown in Figure 4E, a cut-off (<0.9482) for ADAMTS8 could be determined with an 83.7% sensitivity and a 71.4% specificity for separating LC patients from healthy subjects (AUC=0.6877). Additionally, the result of using a logistic regression model to combine the data from the two markers showed that the AUC for combined ROC was significantly improved (AUC=0.75) compared with the AUC for HJURP or ADAMTS8 alone. The results suggested that the abnormality of plasma HJURP and ADAMTS8 levels could be useful molecular markers for LC diagnosis. Also, ROC curve analysis further indicated that there was no significant difference of GPT2 expression between LC patients and healthy controls (AUC=0.5760).

### Clinicopathologic characteristics of candidate biomarkers

The clinicopathologic characteristics of circulating HJURP and ADAMTS8 were shown in Table 1. Interestingly, the lower expression of HJURP was significantly associated with stage IV LC patients (p=0.0422). However, no significant difference was found between HJURP expression and patient sex, age, tumor location, or histology types (p>0.05). Specifically, ADAMTS8 expression tended to be remarkably lower in non-small cell lung cancer group than small cell lung cancer group. Similarly, there was no correlation between ADAMTS8 expression and other characterictics such as age, sex, tumor stage, or tumor location (p>0.05).

**Table 1:** Correlations between HJURP and ADAMTS8 expressions in lung cancer cfRNA and clinicopathologic characteristics

Variables	HJURP			ADAMTS8		
	n	Expression status	P	n	Expression status	P
**Age**						
>60	25	5.54(0.0117-50.00)	0.774	23	5.027(0.0212-38.28)	0.970
≤60	22	0.9423(0.0309-4.828)		20	0.4856(0.0246-5.003)	
**Sex**						
Male	35	4.603(0.0117-50)	0.347	32	3.857(0.0212-38.28)	0.309
Female	12	1.014(0.0514-4.828)		11	0.1725(0.0246-0.6549)	
**Stage**						
I+II+III	11	4.632(0.1211-17.99)	0.042*^a^	9	0.2079(0.0212-0.934)	1
IV	31	3.524(0.0117-50)		29	2.904(0.0246-38.28)	
**Tumor location**						
Left lung	18	1.241(0.0117-14.98)	0.185	16	0.4144(0.0212-5.003)	0.286
Right lung	29	5.205(0.0309-50)		27	4.397(0.0349-38.28)	
**Histology types**						
Non-small cell lung cancer	40	2.481(0.0117-42.11)	0.226	36	1.320(0.0212-27.76)	0.011*^a^
Small cell lung cancer	5	4.058(0.0309-8.319)		9	7.84(0.1338-24.12)	

* a: p value<0.05.

### Transcriptional expression level of HJURP and ADAMTS8 mRNA regarding various tumor entities and coexpression genes

**Figure 5:**
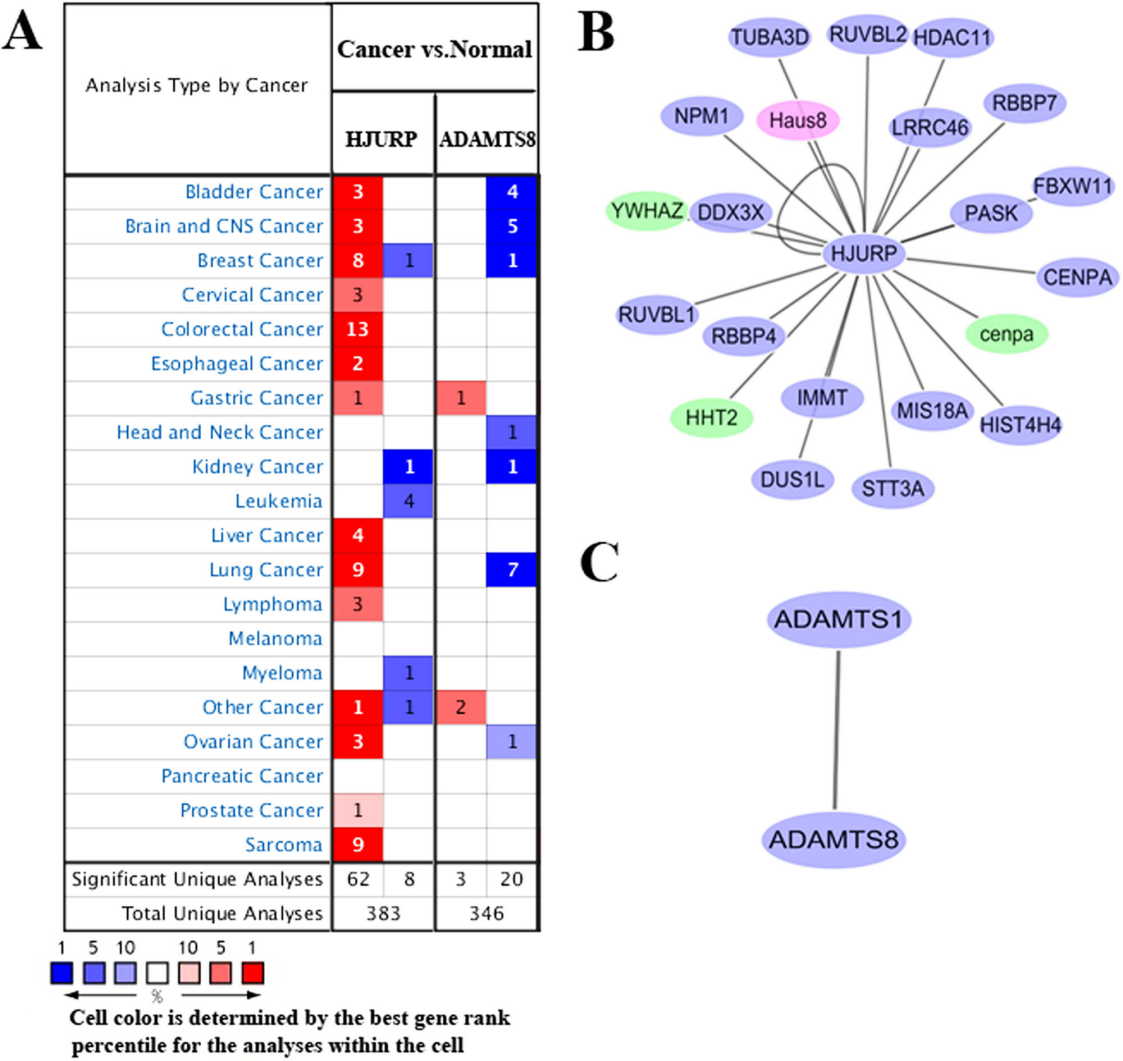
**(A)** Oncomine analysis of HJURP and ADAMTS8 gene expression in normal and cancer tissues of different types. Gene summary views for HJURP and ADAMTS8 genes were shown, which indicated the number of significant unique microarray results with changes>2.0 fold (P<1E-4 by two tailed t-test). The bottom rows in each case indicate the total number of unique analyses and the number of unique analyses that show significant overexpression (red) or underexpression (blue) of the target gene in the cancer samples relative to the normal tissue samples. Genes coexpressed with HJURP **(B)** and ADAMTS8 **(C)** were analyzed using Cytoscape 3.3.0 from different species. Blue node represents gene from Homo species. Red node represents gene from Mus species. Green node represents gene from Bos taurus species.

To further explore the potential roles of circulating HJURP and ADAMTS8 mRNAs in different tumor entities, expression level of HJURP and ADAMTS8 in 20 common cancers was compared with normal samples using Oncomine database. As shown in Figure 5A, expression of HJURP was inconsistent, being overexpressed in 14 of 20 cancer types, especially colorectal cancer, breast cancer, sarcoma as well as lung cancer and underexpressed in 4 types. However, our qPCR assay showed that HJURP expression level was significantly underexpressed in cfRNA of LC patients compared with that of healthy controls. The inconsistency of HJURP expression trend between tissue RNA and plasma circulating mRNA may be due to several reasons, which would be discussed next. Meanwhile, we found that ADAMTS8 was downregulated in a variety of cancers including bladder cancer, brain cancer and lung cancer. In addition, the protein-protein interaction networks for HJURP and ADAMTS8 in the most significant modules among different species were shown in Figure 5B and 5C.

## DISCUSSION

Lung cancer remains associated with significant mortality worldwide. The lack of specific and sensitive biomarkers for early diagnosis as well as targeted therapies contributes to poor overall survival rate. A blood-based biomarker is a cost- and effective tool because blood is easily accessible in a noninvasive manner and measurements could be repeated over time [24]. Recently, numerous research groups have investigated the potential use of circulating mRNA as biomarker for cancer diagnosis [25, 26]. Identification of biomarkers for multiple cancer types can be carried out in various manners. The general experimental strategy for potential circulating mRNA biomarkers discovery is to employ microarray technology, which is then followed by validation using RT-PCR. However, a systematic study deciphering the transcriptional expression of the candidate circulating mRNA biomarkers by integrating diverse microarray data is still lacking. Herein, we describe a novel approach to biomarker discovery that take advantage of underused public data. Meanwhile, we believe through this study that the strategy initially developed for lung cancer can be extended to other kinds of cancer. To our knowledge, this is the first systematic and comprehensive study to identify potential circulating mRNA biomarkers.

RNA was commonly thought to be easily degradable, highly labile and rarely abundant. However, numerous recent studies have demonstrated that RNA released into the circulation is surprisingly stable in spite of the point that increased amounts of RNases circulate in the blood of cancer patients and various kinds of RNA, including mRNA and non-coding RNA can be extracted and detected in the circulating plasma, serum and other bodily fluids [27–30]. RNA protection in the blood may be provided by inclusion into lipoproteic complexes or phospholipids [30]. In addition, the inability to obtain primary tumor tissues, particularly through repeat biopsy, from patients with advanced-stage lung cancer makes the use of plasma as a surrogate fashion for genetic analysis clinically effective. In the present study, we found that the levels of plasmatic HJURP and ADAMTS8 were significantly downregulated in LC patients than in healthy controls, with remarkable diagnostic value for LC. Specifically, HJURP and ADAMTS8 showed statistically significant association with certain clinical aspects of LC. The mechanism accounting for the release of plasma mRNA is not well understood. The most common hypothesis is that they come from apoptosis or necrosis of tumor cells at the tumor site, lysis or apoptosis of circulating tumor cells, or an active release of nucleic acids from the tumor, or even from degenerative normal cells adjacent to tumor cells [31, 32].

HJURP (Holiday Junction Recognizing Protein) located at chromosome 2q37 is a novel tumor suppressor shown to be an independent prognostic of death risk for cancer patients [33]. Recent studies have reported that HJURP is responsible for centromere protein A (CENPA) locationzation for loading of new CENPA nucleosomes during the cell cycle and involved in repairing double-strand DNA breaks [34]. HJURP downregulation resulted in a dramatic loss of CENPA from centromeres and induced genomic instability involving chromosome segregation defect during mitosis and then promote tumorigenesis [35]. Several studies have reported that HJURP overexpression was observed in a majority of lung cancer and breast cancer tissue samples [36]. However, in our study the expression of HJURP was significantly downregulated in cfRNA samples from patients with LC, compared with normal controls. The reasons accounting for the inconsistency could be explained by the differences in the number of patients studied, age, stage (early stage vs. metastatic stage), performance status, molecular characteristics, or other technical variables, including the amount of blood collected, storage, and RT-PCR method (primers, reagents and PCR conditions) [37].

ADAMTS8 located at chromosome 11q24 is a member of the ADAMTS (a disintegrin and metalloprotease with thrombospondin-1-like motifs) family with antiangiogenic property [38]. Unlike conventional ADAM family members, the ADAMTS family members do not have transmembrane regions and are secreted from cells in a catalytically active form. ADAMTS8 is also known to be highly expressed in both adult and fetal lung, and is found at lower levels in the brain, heart, stomach and kidney. Downregulation of ADAMTS8 through epigenetic silencing has been detected in multiple tumors including brain cancer, breast cancer and non-small cell lung cancer [39].

In conclusion, we identified candidate noninvasive biomarkers for lung cancer through bioinformatic analysis of the public Oncomine database and validated their potential using clinical specimens. In addition, our paper is the first study on plasma levels of HJURP and ADAMTS8 mRNA in LC patients. Nevertheless, sample size is a crucial factor to gain statistically significant results. The clinical samples employed in this study were relatively limited. Hence, large-scale studies should be performed to investigate the potential role of HJURP and ADAMTS8 in LC in the future.

## MATERIALS AND METHODS

### Ethics statement

All experimental protocols were approved by the Clinical Research Ethics Committee of the First Affiliated Hospital of Xiamen University. All methods were performed in accordance with the Declaration of Helsinki. Written informed consent was obtained from all human participants after complete description of the study.

### Genome-wide expression analyses by oncomine

A bioinformatics flow was established to analyze lung cancer microarrays in the Oncomine database (Figure 1). Oncomine platform is an industry-standard tool aimed at computing gene expression signatures and extracting biological insights from the database [40]. Differential genome-wide expression analysis on the basis of microarray data comparing the most common types of cancer with their respective normal controls as well as various cancer subtypes are ready to be explored [41]. A simple normalization strategy including a log2 transformation and median centering is employed to all microarray datasets regardless of the platforms, the pre-treatment methods and other variables [42]. For our analysis of cancer vs. normal tissue mRNA, we focused on primary tumors and the following cut-offs were employed p-value≤10^-4^ and fold change≥2. Lists of overexpressed and underexpressed mRNAs in lung cancer were available from each study.

### Clinical specimens

Blood specimens from 47 primary LC patients and 14 healthy subjects were drawn before therapeutic intervention by venapuncture and processed within 2hr. Plasma was collected from the 8 ml blood specimens after centrifugation at 1,600×g for 10 min and 10,000×g for 10 min both at 4°C and stored at -80°C until processing for RNA extraction. Demographic, clinical and histopathological parameters of all these cases were shown in Table 2.

**Table 2:** Clinical characteristics of patients with Lung cancer and healthy controls

Clinicopathologic factors	Serum study	
	LC Cases	Healthy controls
**Total**	47	14
Mean Age±SD	59.23±9.26	43.64±14.91
**Sex**		
Male	35	9
Female	12	5
**Stage**		
I	1	
II	2	
III	8	
IV	31	
NA	5	
**Histology types**		
**Non-small cell lung cancer**		
Adenocarcinoma	26	
Squamous cell carcinoma	13	
Large cell carcinoma	1	
**Small cell lung cancer**	5	
**NA**	2	
**Tumor location**		
Left lung	18	
Right lung	29	

### RNA extraction

RNA was isolated from 2ml plasma using TRIzol LS reagent (cat#10296018, Thermo Fisher Scientific Inc.) according to manufacturer's instructions as previously described [43, 44]. In brief, 2 ml plasma was separated into eight aliquots (250μl), and each aliquot was mixed with 750μl of TRIzol. After 5min incubation at 4°C, 400μl chloroform was added, followed by 30 sec of volient shaking. The mixture was immediately centrifuged at 12,000g for 5min at 4°C. The above aqueous layer was transferred in to a fresh tube containing 800μl isopropyl alcohol. The RNA in aqueous layer was precipitate for 12-16hr at -20°C and washed with 1 ml 75% ethanol. Lastly, the RNA pellet was dried for 3-5min at room temperature, dissolved in 10μl Rnase-free water and then the all aliquots were pooled together. The RNA concentration was quantitated using Qubit RNA HS Assay Kit and Qubit 3.0 fluorometer (ThermoFisher Scientific Inc. cat#Q32851). The concentration of RNA isolated from plasma ranged from 2.12 to 12.26ng/μl.

### Conventional RT-PCR analysis

The extracted total RNA was reverse-transcripted into cDNA using PrimeScript RT reagent Kit (TAKARA cat#RR047A) according to the manufacturer's protocol in triplicates. The resulting cDNA was pooled for next PCR amplification. Primer sequences are shown in Supplementary Table 1. The target sequences were amplified by PCR in 50μl of 1×Taq buffer containing 0.3μM of each primer, 1.5 mM magnesium chloride, 200μM dNTP mixture, 2.5 units of Taq polymerase and 1μl (10μg) of each cDNA by Ex Taq PCR kit (TAKARA cat#RR001A). The reaction was started after 5min denaturation of cDNA at 94°C (hot start). DNA amplification in a T100 cycler (Bio-Rad Laboratories, Inc) was followed by a final extension for 8min at 72°C for HJURP, ADAMTS8 and GPT2 (94°C 30s, 58.5°C 1min; 40 cycles). Although a range of annealing temperatures (from 5°C below the Tm to 5°C above the Tm) had been tried, the PCR products of other 9 mRNAs (TOP2A, GINS2, TK1, CDCA5, AGER, FHL1 CLDN18, ADH1B, and GPIHBP1) were rare compared with positive control. GAPDH expression was used as an internal control. All PCR products were visualized by 2% agarose gels electrophoresis. Water negative controls contained all components for the RT-PCR reaction without target RNA. Positive controls of RNA were extracted from A594 cells obtained from the Cancer Center of Xiamen University (Xiamen, China).

### Quantitative PCR (qPCR)

For further validation, qPCR was carried out in duplicate at 50°C for 2min, denaturing at 95°C for 5min, followed by 40 cycles of 95°C for 30s, 58.5°C for 1min by using ABI ViiA 7 Real-Time PCR System (Applied Biosystems) with melting curve analysis. GAPDH was employed as a reference gene applying the 2^–ΔCT^ algorithm (ΔCT=Ct. target-Ct. reference) [45].

### Statistical analysis

Kolmogorov–Smirnov test was applied to determine the distribution of the samples of each group. Data were showed as median and range. Expression levels of plasma cell-free mRNAs between two groups were compared using the Mann–Whitney U test. The ratios of qPCR expression values of normal and cancer samples were used after normalization by the expression value of internal control GAPDH gene. The association between relevant gene expression levels and clinical parameters was addressed by Mann–Whitney U test. The diagnostic performance of HJURP and ADAMTS8 was identified using receiver operating characteristic (ROC) curves and the area under the curve (AUC). Cut-offs for the two biomarkers were estimated at various sensitivities and specificities and at the maximum Youden's index (sensitivity+specificity–1) [46]. Moreover, the two markers were multiplied by their logistic regression coefficient and added to give a combined AUC value. Statistical analyses were carried out using GraphPad Prism 7.0 (GraphPad Soft-ware Inc., La Jolla, CA, USA) and MedScalc software. Summary data were reported as mean±SEM. A p value of less than 0.05 was considered statistically significant.

## SUPPLEMENTARY MATERIALS TABLES


