# Supplementary material for: Clinical verification of plasma messenger RNA as novel noninvasive biomarker identified through bioinformatics analysis for lung cancer
Source: Oncotarget. 2017 Mar 30;8(27):43978–89. doi: 10.18632/oncotarget.16701 (PMC5546455; doi:10.18632/oncotarget.16701)
Supplement: Supplementary file 1 [file oncotarget-08-43978-s001.pdf]

Clinical verification of plasma messenger RNA as novel noninvasive biomarker identified through bioinformatics analysis for lung cancer

SUPPLEMENTARY TABLE

Supplementary Table 1: Primers used and PCR conditions

Primer	Sequence	Length of PCR product (bp)	Annealing temperature (ºC)
TOP2A	ggtgtggaactagaaggcct<br>tctgtttctcgtggagggac	194	59
GPT2	cagaggaggctacatggagg<br>ccttctctcggctgaattgc	173	58.5
GINS2	aatgcccagcccttactaca<br>cctgctgtctcacaaagctg	161	59
HJURP	ctgcccaagagcgattcatc<br>gtaacgattccttccgtggc	189	58.5
TK1	aattgtggctgcactggatg<br>agtggtacttgtctgctccc	191	59
CDCA5	agggccccatctcctactaa<br>gacagctgggacctctacag	168	59
AGER	ctaccgagtccgtgtctacc<br>ttctcattaggcaccagggg	171	59
FHL1	gaagtgcaacaaggccatca<br>gtgatggggttcttgcatcc	198	59
CLDN18	ccgaatgcaggccctatttc<br>gaggtcagtgtcatgttggc	181	59
ADAMTS8	agagcagcaaagagagagca<br>ggtttcagagccttgttgca	178	58.5
ADH1B	ttggctgtggattctcgact<br>cttgttgatgtccaccgcaa	164	59
GPIHBP1	ggatgaggtggaagaggagg<br>caatgagggttgtgcaggtc	152	59
